# Single-Cell Atlas Reveals Fatty Acid Metabolites Regulate the Functional Heterogeneity of Mesenchymal Stem Cells

**DOI:** 10.3389/fcell.2021.653308

**Published:** 2021-04-12

**Authors:** Jiayi Xie, Qi Lou, Yunxin Zeng, Yingying Liang, Siyu Xie, Quanhui Xu, Lisha Yuan, Jin Wang, Linjia Jiang, Lisha Mou, Dongjun Lin, Meng Zhao

**Affiliations:** ^1^Department of Hematology, The Seventh Affiliated Hospital, Sun Yat-sen University, Shenzhen, China; ^2^Shenzhen Lansi Institute of Artificial Intelligence in Medicine, Shenzhen, China; ^3^The First Affiliated Hospital of Shenzhen University, Health Science Center, Shenzhen Second People’s Hospital, Shenzhen, China; ^4^RNA Biomedical Institute, Sun Yat-sen Memorial Hospital, Sun Yat-sen University, Guangzhou, China; ^5^Key Laboratory of Stem Cells and Tissue Engineering, Zhongshan School of Medicine, Sun Yat-sen University, Ministry of Education, Guangzhou, China

**Keywords:** single-cell RNA-seq, butyrate, cell heterogeneity, HSC niche, mesenchymal stem cells

## Abstract

Bone marrow mesenchymal stem cells (MSCs) are widely used clinically due to their versatile roles in multipotency, immunomodulation, and hematopoietic stem cell (HSC) niche function. However, cellular heterogeneity limits MSCs in the consistency and efficacy of their clinical applications. Metabolism regulates stem cell function and fate decision; however, how metabolites regulate the functional heterogeneity of MSCs remains elusive. Here, using single-cell RNA sequencing, we discovered that fatty acid pathways are involved in the regulation of lineage commitment and functional heterogeneity of MSCs. Functional assays showed that a fatty acid metabolite, butyrate, suppressed the self-renewal, adipogenesis, and osteogenesis differentiation potential of MSCs with increased apoptosis. Conversely, butyrate supplement significantly promoted HSC niche factor expression in MSCs, which suggests that butyrate supplement may provide a therapeutic approach to enhance their HSC niche function. Overall, our work demonstrates that metabolites are essential to regulate the functional heterogeneity of MSCs.

## Introduction

Mesenchymal stem cells (MSCs) are multipotent fibroblast colony-forming cells, which can differentiate into adipocytes, chondrocytes, and osteocytes (Friedenstein et al., 1974; Horwitz et al., 2005; Uccelli et al., 2008; Bianco et al., 2013; Zhou et al., 2014). Bone marrow MSCs generate osteoblasts and provide the major source of bone formation during development and regeneration after bone damage (Kassem et al., 2008; Ye et al., 2012; Valenti et al., 2016; Pajarinen et al., 2019). Furthermore, bone marrow MSCs secrete multiple growth factors to support hematopoietic stem cells (HSCs) for their maintenance and regeneration (Mendez-Ferrer et al., 2010; Zhou et al., 2017). Recent works show that bone marrow MSCs also contribute to immunomodulation against infection and autoimmune diseases as well as tissue repair, such as skin and blood vessels (Sun et al., 2009; Zhang et al., 2015; Shook et al., 2020).

The versatile functions of MSCs enable increasing clinical applications of MSCs to treat diseases such as graft versus host disease (GvHD), Crohn’s disease, heart failure, bone marrow failure syndrome, and osteogenesis imperfecta and bone fractures (Galipeau and Sensebe, 2018; Andrzejewska et al., 2019; Martin et al., 2019; Yin et al., 2019). MSC infusion is also applied for facilitating implantation after HSC transplantation (Mendez-Ferrer et al., 2010; Zhou et al., 2017). However, the diverse therapeutic targets challenge the clinical trials of MSCs in which the cell functional heterogenicity, culture methods, and expansion levels could potentially influence the therapeutic consistency and limit clinic efficacy of MSCs (Pattappa et al., 2013; Moll et al., 2014; Yuan et al., 2019).

The cellular metabolism profile controls stem cell fates in self-renewal, lineage commitment, and terminal differentiation (Ryall et al., 2015a; Teslaa and Teitell, 2015; Garcia-Prat et al., 2017). Metabolites, such as acetyl-coenzyme A (Wang et al., 2009), α-ketoglutarate (Hwang et al., 2016), S-adenosylmethionine, and S-adenosylhomocysteine (Shyh-Chang et al., 2013), regulate the proliferation and differentiation of embryonic stem cells (ESCs). The successful reprogramming of induced pluripotent stem cells (iPSCs) requires a metabolic shift from oxidative phosphorylation to anaerobic glycolysis (Yoshida et al., 2009; Folmes et al., 2011). Muscle stem cells reside in an aerobic niche near capillaries (Christov et al., 2007), and transition into committed progenitors is accompanied by a switch from fatty acid (FA) oxidation to glycolysis (Ryall et al., 2015b). Furthermore, metabolic status regulates MSCs in cell fate determination and multifunction maintenance. Bone marrow MSCs reside in a hypoxic niche and rely on anaerobic glycolysis to maintain their self-renewal and multipotency and evade senescence (Ito and Suda, 2014). Glutaminase, the key enzyme in glutamine metabolism, and 5-methoxytryptophan, a tryptophan metabolite, promote osteogenic and suppress adipogenic MSC differentiation (Chang et al., 2017; Yu et al., 2019). On the contrary, arachidonic FA induces MSC adipogenesis but inhibits osteogenesis in human MSC cultures (Casado-Diaz et al., 2013). Unsaturated FAs, such as linoleic and oleic acids, inhibit MSC proliferation and induce the expression of angiogenesis mediators, such as IL-6, IL-8, VEGF, and nitric oxide (Smith et al., 2012).

Recent works show that butyrate, a natural short-chain fatty acid (SCFA) produced by mammalian intestinal microbiota, can inhibit histone deacetylase (HDAC) activity and impairs intestinal epithelial stem/progenitors in wound repair *in vivo* (Kaiko et al., 2016) but promotes iPSC reprogramming efficiency (Liang et al., 2010). However, the role of butyrate in regulating MSCs remains elusive. Here, using single-cell RNA sequencing (scRNA-seq), we identified FA pathways that are involved in lineage commitment of MSCs, and further functional assays prove that metabolite butyrate alters MSC cell fate in self-renewal, apoptosis, and HSC niche factor expression.

## Materials and Methods

### Mice

C57BL/6 mice were obtained from the Jackson Laboratory and were maintained in the C57BL/6 background. All animal experiments were performed according to protocols approved by the institutional animal care and use committee.

### Bone Marrow Digestion

Bone marrow digestion was performed as described with small changes (Zhou et al., 2014). In brief, intact bone marrow from mice at the age of 6–8 weeks were flushed from mouse femora and tibiae and subjected to two rounds of enzymatic digestion at 37°C for 20 min each. The digestion buffer contained 0.2 mg/ml liberase (Roche) and 200 μg/ml DNAse I (Roche) in 1 × HBSS with calcium and magnesium. The digested marrow cells then underwent red blood cell lysis using 0.16 M ammonium chloride solution.

### Flow Cytometry and Cell Sorting

For cell sorting and analysis, monoclonal antibodies to CD45 (30-F11, Biolegend), Ter-119 (TER-119, eBioscience), PDGFRα (APA5, eBioscience), CD31 (MEC13.3, Biolegend), CD51 (RMV-7, Biolegend), and 7AAD (Biolegend) were used where indicated. Cell sorting was performed using a cell sorter (MoFlo Astrios). Cell analysis was performed on a flow cytometer (Attune NxT, Thermo Fisher).

### scRNA-seq

Sorted CD45^–^Ter-119^–^CD31^–^PDGFα^+^CD51^+^ single cells were processed through the Chromium Single Cell Platform using the Chromium Single Cell 3′ Library and Gel Bead Kit v3 (10X Genomics, PN-1000075) and the Chromium Single Cell B Chip Kit (10X Genomics, PN-1000074) as the manufacturer’s protocol. In brief, 15,000 cells were loaded onto the chromium instrument to generate single-cell barcoded droplets. Cells were lysed and barcoded reverse transcription of RNA was occurred. Libraries were prepared by following the amplification, fragmentation, adaptor, and index attachment and then sequenced on an Illumina NovaSeq platform. The scRNA-seq data generated in this study are deposited in GEO (GSE167035^1^).

### scRNA-seq Data Processing

The scRNA-seq reads were aligned to the mm10 reference genomes, and unique molecular identifier (UMI) counts were obtained by CellRanger 3.0.2. Normalization, dimensionality reduction, and clustering were performed with the Seurat 3.0 R package (Butler et al., 2018) on RStudio, and 3005 of 11,888 cells with *Ptprc* (CD45) expression were excluded to remove potential contamination. Cells were filtered to have > 200 and < 7000 detected genes and less than 5% of total UMIs mapping to the mitochondrial genome. Data set normalization was performed by dividing the UMI counts per gene by the total UMI counts in the corresponding cell and log-transforming, followed by the results scaling and centering. Cells underwent dimensionality reduction with the t-stochastic neighboring embedding (tSNE) method and partition-based graph abstraction (PAGA) using scanpy (Wolf et al., 2018). Integration of published MSC and immune cell scRNA-seq with our data was performed by Seurat with the function SCTransform() and Harmony algorithm. Signature genes of each cluster were obtained using the Seurat function FindMarkers with the Wilcox test. All correlations were calculated based on values with the function cor() and the parameter “method = ‘spearman’.” The pseudotime trajectory was analyzed by monocle2 based on the Seurat clustering (Qiu et al., 2017). A signature gene heat map was generated by pheatmap R packages. GOChord plots and GOClust plots were generated by GOplot R packages. Gene set enrichment analysis (GSEA) was performed using the gsea R package (Subramanian et al., 2005). Gene lists were preranked by the fold change values of the differential expression analysis using Seurat. GSEA plots were generated by enrichplot R packages. Gene sets were obtained from the gene ontology database as indicated. Signature genes feature plots and violin plots were generated with Seurat R packages.

### CFU-F Assay and MSC *in vitro* Differentiation Assay

For the CFU-F assay, freshly isolated marrow cells were plated at a density of 5 × 10^5^ cells/well in six-well plates with DMEM (Corning) plus 20% fetal bovine serum (Gibco), 10 μM ROCK inhibitor (Selleck), and 1% penicillin/streptomycin (Hyclone). Cell cultures were maintained at 37°C, 5% O_2_, and 5% CO_2_ chambers. CFU-F colonies were counted after 7 days of culture by staining with crystal violet (Sangon). For the *in vitro* differentiation assay, we enzyme-digested CFU-Fs and subcloned them into six-well plates at a density of 1 × 10^5^ cells/well. Cells then underwent adipogenic (7 days) or osteogenic (14 days) differentiation with StemPro Differentiation Kits (Gibco). Adipogenic differentiation was quantified by Oil Red O staining (Sigma). Osteogenic differentiation was quantified by Alizarin Red S (Sigma). In indicated groups, butyrate (500 nM, 5 μM, and 500 μM as indicated), SAHA (Vorinostat, 1 μM), Z-VAD-FMK (30 μM), or Necrostatin-1 (45 μM) were supplemented into the culture medium.

### qPCR

For qPCR, CFU cells were dissociated in Trizol (Magen), and RNA was extracted following the manufacture’s instruction. RNA was reverse transcribed into cDNA using the TransScript All-in-One First-Strand cDNA Synthesis kit (Transgene). Quantitative PCR was performed using a Bio-Rad CFX 96 touch. The primers for *Runx2* were 5′ GACTGTGGTTACCGTCATGGC 3′ (forward) and 5′ ACTTGGTTTTTCATAACAGCGGA 3′ (reverse). The primers for *Ocn* were 5′ CAGACACCATGAGGAC CATC 3′ (forward) and 5′ GGACTGAGGCTCTGTGAGT 3′ (reverse). The primers for *Col1a1* were 5′ GCTCCTCTTAGGG GCCACT 3′ (forward) and 5′ ATTGGGGACCCTTAGGCCAT 3′ (reverse). The primers for *Fabp4* were 5′ AAGGTGAAGA GCATCATAACCCT 3′ (forward) and 5′ TCACGCCTTTC ATAACACATTCC 3′ (reverse). The primers for *Adiponectin* were 5′ TGTTCCTCTTAATCCTGCCCA 3′ (forward) and 5′ CCAACCTGCACAAGTTCCCTT 3′ (reverse). The primers for *Cebpa* were 5′ GCGGGAACGCAACAACATC 3′ (forward) and 5′ GTCACTGGTCAACTCCAGCAC 3′ (reverse). The primers for *Cmyc* were 5′ ATGCCCCTCAACGTGAACTTC 3′ (forward) and 5′ GTCGCAGATGAAATAGGGCTG 3′ (reverse). The primers for *Ccnb1* were 5′ AAGGTGCCTGT GTGTGAACC 3′ (forward) and 5′ GTCAGCCCCATCATCT GCG 3′ (reverse). The primers for *Ccnd1* were 5′ GCGTACC CTGACACCAATCTC 3′ (forward) and 5′ CTCCTCTTCGCAC TTCTGCTC 3′ (reverse). The primers for *Bcl2* were 5′ GTCG CTACCGTCGTGACTTC 3′ (forward) and 5′ CAGACATGCA CCTACCCAGC 3′ (reverse). The primers for *Bax* were 5′ TG AAGACAGGGGCCTTTTTG 3′ (forward) and AATTCGCCG GAGACAC TCG 3′ (reverse). The primers for *Bak1* were 5′ CAACCCCGAGATGGACAACTT 3′ (forward) and 5′ CGTAG CGCCGGTTAATATCAT 3′ (reverse). The primers for *Bid* were 5′ GCCGAGCACATCACAGACC 3′ (forward) and 5′ TGGCAATGTTGTGGATGATTTCT 3′ (reverse). The primers for *Kitl* were 5′ AGGAACGGAACAGAAAGG 3′ (forward) and 5′ GTCGGATAGACTTCACTTGG 3′ (reverse). The primers for *Angpt1* were 5′ CACATAGGGTGCAGCAACCA 3′ (forward) and 5′ CGTCGTGTTCTGAAGAATGA 3′ (reverse). The primers for *Cxcl12* were 5′ AGGTTCTTATTTCACGG CTTGT 3′ (forward) and 5′ TGGGTGCTGAGACCTTTGAT 3′ (reverse). The primers for *Jag2* were 5′ CAATGACACCACTC CAGATGAG 3′ (forward) and 5′ GGCCAAAGAAGTCGT TGCG 3′ (reverse).

### Cell Counting Kit-8 (CCK-8) and Lactate Dehydrogenase (LDH) Activity Assay

Freshly isolated marrow cells were plated at a density of 2 × 10^4^ cells/well in a 96-well plate and cultured with or without butyrate, ZVAD, or Nec-1 as indicated for 24 h in 37°C, 5% O_2_, and 5% CO_2_ chambers. For the CCK-8 assay, 10 μl CCK-8 reagent (Solarbio) was added in wells and continued incubated for 4 h in chambers. The optical density was measured at 450 nm using a microplate reader. LDH activities were performed following the manufacturer’s instructions (Njjcbio). Cell death frequency was calculated as LDH release into cell culture medium dividing LDH in the total cell lysate.

### Statistical Analyses

Data are presented as mean ± s.e.m. For multiple comparisons analysis, data were analyzed by repeated-measures one-way ANOVA followed by Dunnett’s test. The difference was considered statistically significant if *p* < 0.05, and 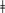
*P* < 0.05, 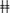
*P* < 0.01, 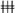
*P* < 0.001. For other experiments, except for scRNA-seq analysis, data were analyzed by two-tailed Student’s *t* test. The difference was considered statistically significant if *p* < 0.05, and ^∗^*p* < 0.05, ^∗∗^*p* < 0.01, ^∗∗∗^*p* < 0.001.

## Results

### Single-Cell Atlas Identifies the Heterogeneity of MSCs

**FIGURE 1:**
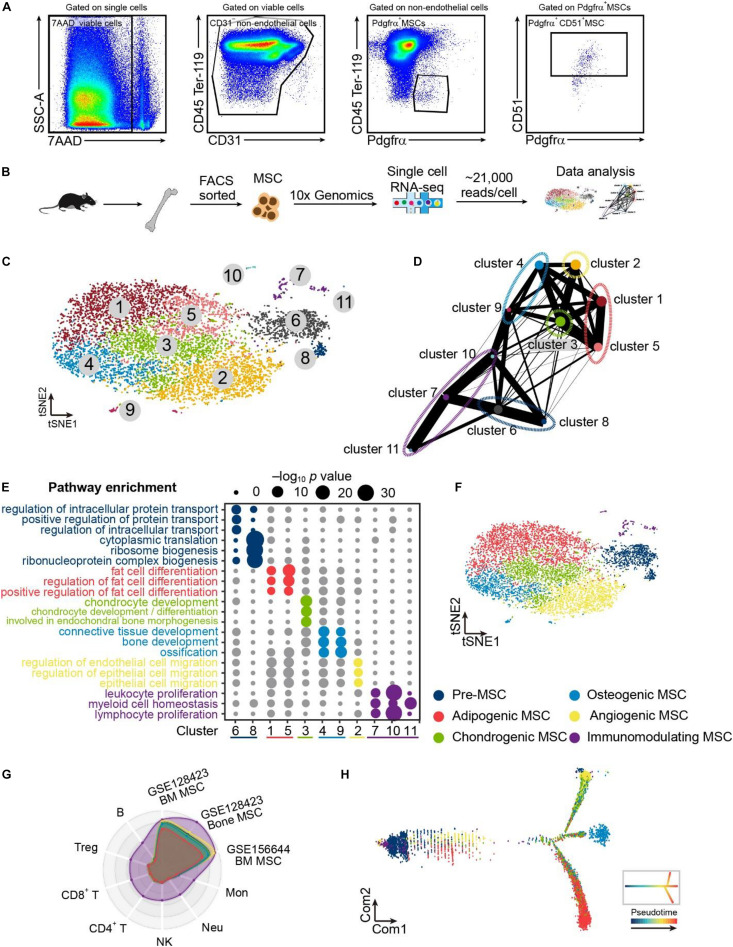
Single-cell atlas identifies heterogeneous MSC populations. **(A)** Flow cytometry gating for isolating bone marrow MSCs. **(B)** Schematic depicting the strategy of cell isolation, MSC sorting, single-cell RNA sequencing, and data analysis. **(C)** Clustering 8883 cells from MSCs by tSNE. **(D)** Results of PAGA. Each node represents a cluster, and edges show the connectivity between clusters. The size of nodes indicates the number of cells in each cluster, and the thickness of the edges denotes the connectivity from low (thin) to high (thick). **(E)** Eleven clusters annotating by gene sets of GO terms based on their signatures and dividing into pre-, adipogenic, chondrogenic, osteogenic, angiogenic, and immunomodulating MSCs. **(F)** Clustering 8883 cells from bone marrow MSCs annotating six populations inferred from PAGA results and enriched pathways of marker genes. **(G)** Radar chart showing the spearman rho between the MSC scRNA-seq result in this study compared with the published MSC and immune cell scRNA-seq results. Each ring represents one population in **(F)**. **(H)** Developmental pseudotime of MSCs. The inset depicts the schema of pseudotime of MSCs.

To explore the heterogeneity of bone marrow MSCs, we applied droplet-based scRNA-seq with non-hematopoietic (CD45^–^Ter-119^–^), non-endothelial (CD31^–^) bone marrow cells that express MSC markers (PDGFα^+^CD51^+^) (Morikawa et al., 2009; Yue et al., 2016; Boulais et al., 2018; Lee et al., 2018) (Figures 1A,B). We successfully detected a total of 8883 cells with an average of ∼2000 genes per cell in bone marrow MSCs (Supplementary Figure 1A). We then employed t-SNE and identified 11 clusters in MSCs (Figure 1C). Results of PAGA (Figure 1D) and enriched gene ontology (GO) (Figure 1E) annotated the 11 clusters into six populations, including pre-MSCs, adipogenic MSCs, chondrogenic MSCs, osteogenic cells, angiogenic MSCs, and immunomodulating MSCs (Figure 1F). Pre-MSCs, constituted of clusters 6 and 8, enriched pathways in protein transport, nuclear transport, and ribosome biogenesis, which are critical for MSC lineage commitment (Herencia et al., 2012; Chen et al., 2015). Adipogenic MSCs, chondrogenic MSCs, and osteogenic cells, constituted of clusters 1 and 5, cluster 3, and clusters 4 and 9, enriched pathways in fat cell differentiation, chondrocyte development, and bone development, respectively. Angiogenic MSCs were constituted of cluster 2, enriching pathways in endothelial and epithelial cell migration. Immunomodulating MSCs were constituted by clusters 7, 10, and 11, which enriched pathways associated with leukocyte proliferation and myeloid cell homeostasis (Figure 1E). Furthermore, these immunomodulating MSCs overlapped with other MSC subpopulations and published bone marrow MSCs (Baryawno et al., 2019; Leimkuhler et al., 2020) but not with immune cells (Choi et al., 2019) (Figure 1G). In line with this, immunomodulating MSCs did not express any immune cell markers, such as T cells, B cells, NK cells, and macrophages (Supplementary Figure 1B). This ruled out the potential contamination of immune cells in this immunomodulating MSC population. Furthermore, adipogenic MSCs, chondrogenic MSCs, and osteogenic cells were referred to as lineage-committed MSCs. Consistently, our unsupervised trajectory analysis by Monocle 2 showed that the pre-MSCs clustered in the root of the trajectory, and adipogenic, chondrogenic, osteogenic, and angiogenic MSCs clustered in different branches (Figure 1H).

Overall, using scRNA-seq, we identified pre-MSCs and lineage-committed MSC clusters and revealed their potential regulatory mechanisms in MSC lineage commitment.

### Single-Cell Atlas Identifies Pre-MSCs and Lineage-Committed MSCs

**FIGURE 2:**
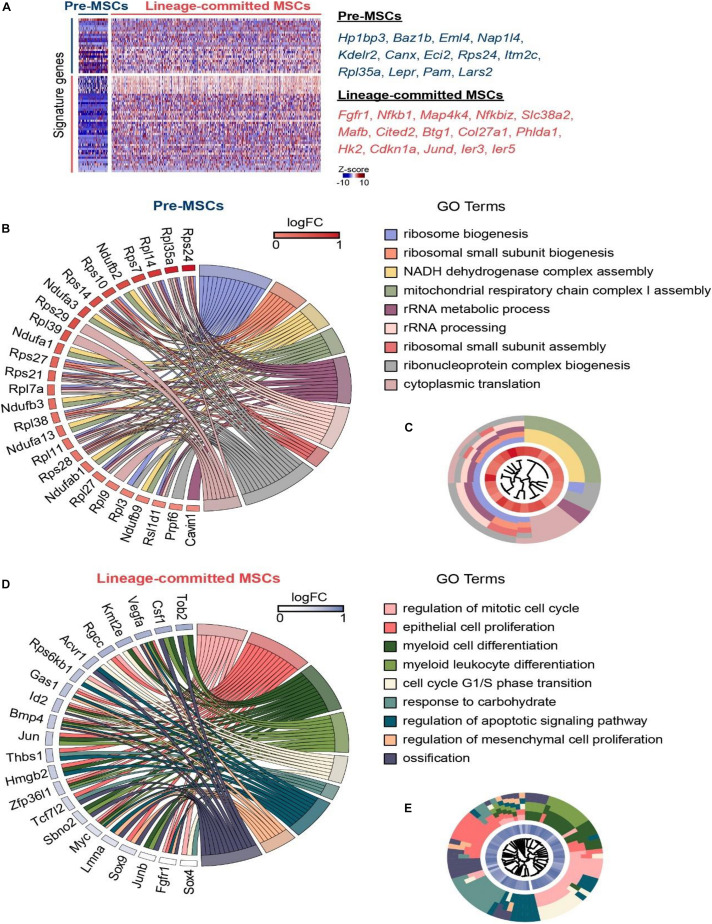
Single-cell atlas identifies pre-MSCs and lineage-committed MSCs. **(A)** Heat map of signature genes in pre- or lineage-committed MSCs; exemplar genes are listed on right. Columns denote cells; rows denote genes. **(B)** GOChord plot showing the association between statistically significant genes in pre-MSCs and their associated GO terms; the genes are associated via ribbons to their assigned terms. White-to-red coding next to the selected genes indicates the gene expression fold change. The outer ring shows the assigned functional terms. **(C)** GOCluster plot showing the clustering of the pre-MSC signature gene expression profiles. The inner ring shows the color-coded expression fold changes, and the outer ring shows the assigned functional terms as indicated in **(B)**. **(D)** GOChord plot showing the association between statistically significant genes in lineage-committed MSCs and their associated GO terms; the genes are associated via ribbons to their assigned terms. White-to-blue coding next to the selected genes indicates the gene expression fold change. The outer ring shows the assigned functional terms. **(E)** GOCluster plot showing the clustering of the lineage-committed MSC signature gene expression profiles. The inner ring shows the color-coded expression fold changes, and the outer ring shows the assigned functional terms as indicated in **(D)**.

Our scRNA-seq data shows that pre-MSCs significantly enriched stemness genes (such as *Hp1bp3* and *Baz1b*) and FA metabolic genes (such as *Eci2* and *Pam*) (Satani et al., 2003; Dutta et al., 2014; van Weeghel et al., 2014; Zanella et al., 2019). Conversely, lineage-committed MSCs dominantly enriched differentiation genes (such as *Col27a1* and *Jund*) and proliferation genes (such as *Fgfr1* and *Mafb*) (Naito et al., 2005; Naba et al., 2017; Ardizzone et al., 2020; Guo et al., 2020) (Figure 2A). This is consistent with previous reports that multipotent MSCs are quiescent (Mendez-Ferrer et al., 2010; Zhou et al., 2014). We further found that pre-MSCs highly enriched ribosome biogenesis and cellular respiration-associated genes, such as *Rps24*, *Rpl35a*, and *Ndufb3* (Choesmel et al., 2008; Narla et al., 2011; Alston et al., 2016) (Figures 2B,C). These indicate that energy metabolism and protein synthesis control are essential for stem cell maintenance (Teslaa and Teitell, 2015; Wanet et al., 2015; Blanco et al., 2016; Sanchez et al., 2016). However, lineage-committed MSCs highly enriched proliferation, lineage commitment, and apoptosis genes, such as *Kmt2e*, *Sox9*, and *Acvr1* (Chakkalakal et al., 2012; Zhang et al., 2017; Liu C.F. et al., 2018) (Figures 2D,E).

Overall, these findings indicate the distinguished regulation mechanisms for multipotent pre-MSCs and lineage-committed MSCs.

### The FA Metabolic Process Regulates MSC Lineage Commitment

**FIGURE 3:**
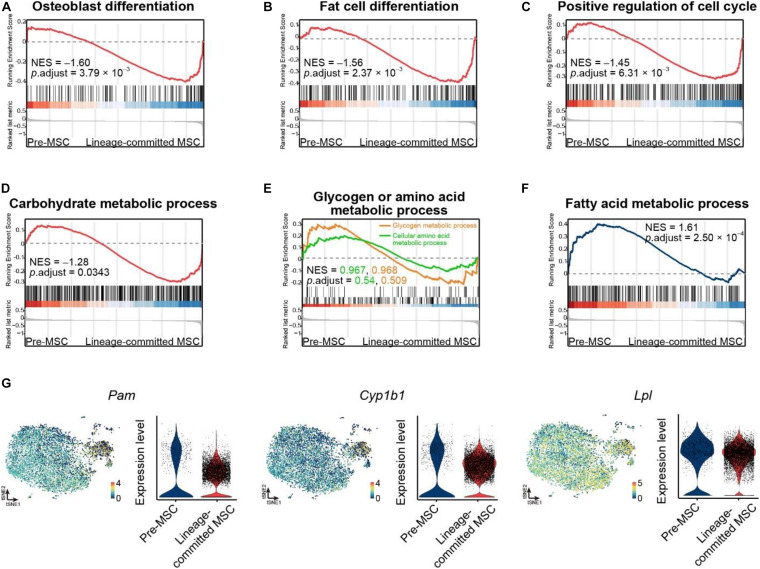
The FA metabolic process regulates MSC lineage commitment. **(A,B)** GSEA evaluating enrichment of osteoblast **(A)** or fat cell differentiation genes **(B)** in pre- and lineage-committed MSCs. Normalized enrichment score and adjusted *p*-value were calculated by permutation tests. **(C)** GSEA evaluating enrichment of cell cycle genes in pre- and lineage-committed MSCs. Normalized enrichment score and adjusted *p*-value were calculated by permutation tests. **(D,E)** GSEA evaluating enrichment of carbohydrate **(D)**, glycogen, and amino acid metabolic process genes **(E)** in pre- and lineage-committed MSCs. Normalized enrichment score and adjusted *p* value were calculated by permutation tests. **(F)** GSEA evaluating enrichment of FA metabolic process genes in pre- and lineage-committed MSCs. Normalized enrichment score and adjusted *p*-value were calculated by permutation tests. **(G)** Feature plots and violin plots showing the selected fatty acid metabolic process genes expression in 8883 cells.

To explore the underlying mechanism in regulating different MSC clusters, we performed GSEA on our scRNA-seq data between pre-MSCs and lineage-committed MSCs using gene sets in the GO database (Ashburner et al., 2000; The Gene Ontology Consortium, 2019). As expected, pre-MSCs had much less osteoblast cell differentiation (GO: 0001649) and fat cell differentiation (GO: 0045444) genes compared with lineage-committed MSCs (NES = –1.60 and –1.56, respectively, Figures 3A,B). Furthermore, we discovered that pre-MSCs are relatively quiescent as they had much less activated cell cycle (GO: 0045787) genes (NES = –1.45, Figure 3C). We next investigated how metabolites regulate pre-MSCs and lineage-committed MSCs in their stem cell fate decision. The GSEAs showed that pre-MSCs had less activated genes under the carbohydrate metabolic process (GO: 0005975) compared with lineage-committed MSCs (NES = –1.28) but were not significantly different under glycogen (GO: 0005977) or amino acid metabolic process (GO: 0006520) (Figures 3D,E). However, pre-MSCs significantly enriched genes under the FA metabolic process (GO: 0006631) (NES = 1.61, Figure 3F). We also confirmed that FA metabolic genes are much enriched in pre-MSCs compared with lineage-committed MSCs, such as *Pam*, *Cyp1b1*, and *Lpl* (Satani et al., 2003; Rozovski et al., 2015; Bushkofsky et al., 2016) (Figure 3G). These indicate that the FA metabolic process might contribute to the regulation of pre-MSCs and lineage-committed MSCs through their distinguished metabolic profile.

Overall, our scRNA-seq data indicate a novel potential role of MSCs responding to FA treatment, which could be applied to *in vitro* culture.

### Supplement of Butyrate Suppresses Self-Renewal and Differentiation Potential of Bone Marrow MSCs

**FIGURE 4:**
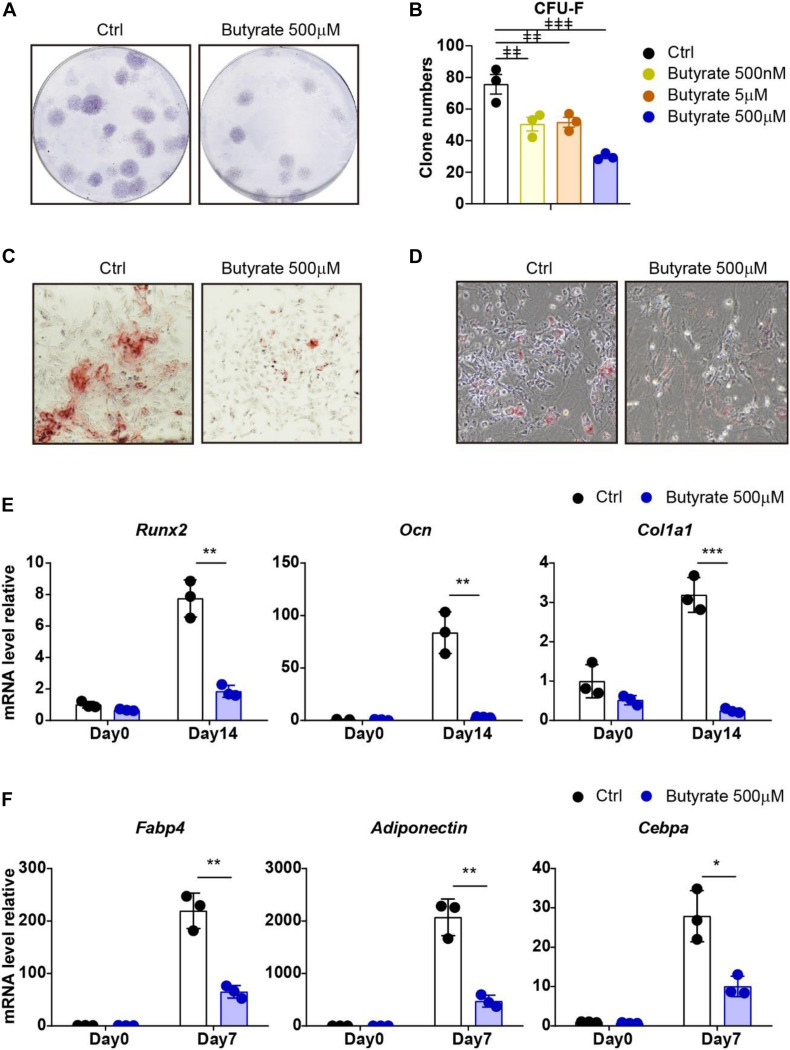
Supplement of butyrate suppresses self-renewal and differentiation potential of MSCs. **(A)** Representative wells of CFU-F colonies stained with crystal violet (5 × 10^5^ bone marrow cells were initially plated). **(B)** CFU-F colony numbers after butyrate treatment as indicated doses or with vehicle control treatment as indicated (5 × 10^5^ bone marrow cells were initially plated). **(C)** Osteogenic differentiation of MSCs with or without butyrate treatment. Alizarin red S staining of MSCs cultured in osteogenic differentiation medium at day 14. **(D)** Adipogenic differentiation of MSCs with or without butyrate treatment. Oil Red O staining of MSCs cultured in adipogenic differentiation medium at day 7. **(E)** Quantitative RT-PCR of transcript levels of osteogenic genes (normalized to day 0 control group). **(F)** Quantitative RT-PCR of transcript levels of adipogenic genes (normalized to day 0 control group). Repeated-measures one-way ANOVA followed by Dunnett’s test for multiple comparisons in **(B)**. 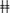
*p* < 0.01, 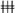
*p* < 0.001. Two-tailed Student’s *t* tests were used to assess statistical significance in **(E,F)**. *p < 0.05, **p < 0.01, ***p < 0.001.

To investigate whether supplement butyrate influences the maintenance of MSCs during *in vitro* culture, we performed a bone marrow MSC CFU-F assay and found that supplement of butyrate at biological serum concentration (500 nM) (Tyagi et al., 2018) or higher concentration (5 μM and 500 μM) effectively reduced the CFU-F clone numbers compared with vehicle control (33.5%, 31.7%, and 60.8% decreased, respectively; Figures 4A,B). These show that butyrate suppressed MSC proliferation and self-renewal potential.

We next asked whether butyrate impairs the self-renewal potential of MSCs due to enhanced differentiation. To this aim, we performed an *ex vivo* differentiation assay to examine the osteogenesis and adipogenesis capacities of MSCs. Interestingly, butyrate-treated MSCs have reduced osteogenic and adipogenic differentiation ability (Figures 4C,D). Consistently, the block of osteogenic differentiation ability was confirmed by multiple osteogenic-specific marker genes, such as *Runx2*, *Ocn*, and *Col1a1*, which were markedly increased after osteogenic differentiation (7.75-fold, 83.7-fold, and 3.19-fold increased, respectively, compared with undifferentiated MSCs) in control MSCs but were inhibited in butyrate-treated MSCs (76.1%, 96.2%, and 92.3% decreased, respectively, compared with differentiated control MSCs, Figures 4C,E). Furthermore, the adipogenic-specific marker genes, such as *Fabp4*, *Adiponectin*, and *Cebpa*, were increased in control MSCs (219-fold, 2071-fold, and 27.9-fold increased, respectively, compared with undifferentiated MSCs) but were dramatically compromised in butyrate-treated MSCs (70.3%, 77.2%, and 64.0% decreased, respectively, compared with differentiated control MSCs; Figures 4D,F).

Overall, our data show that supplement of butyrate during *in vitro* culture impaired MSCs in their self-renewal potential and differentiation abilities.

### Supplement of Butyrate Triggers Apoptosis but Promotes HSC Niche Factor Expression in Bone Marrow MSCs

**FIGURE 5:**
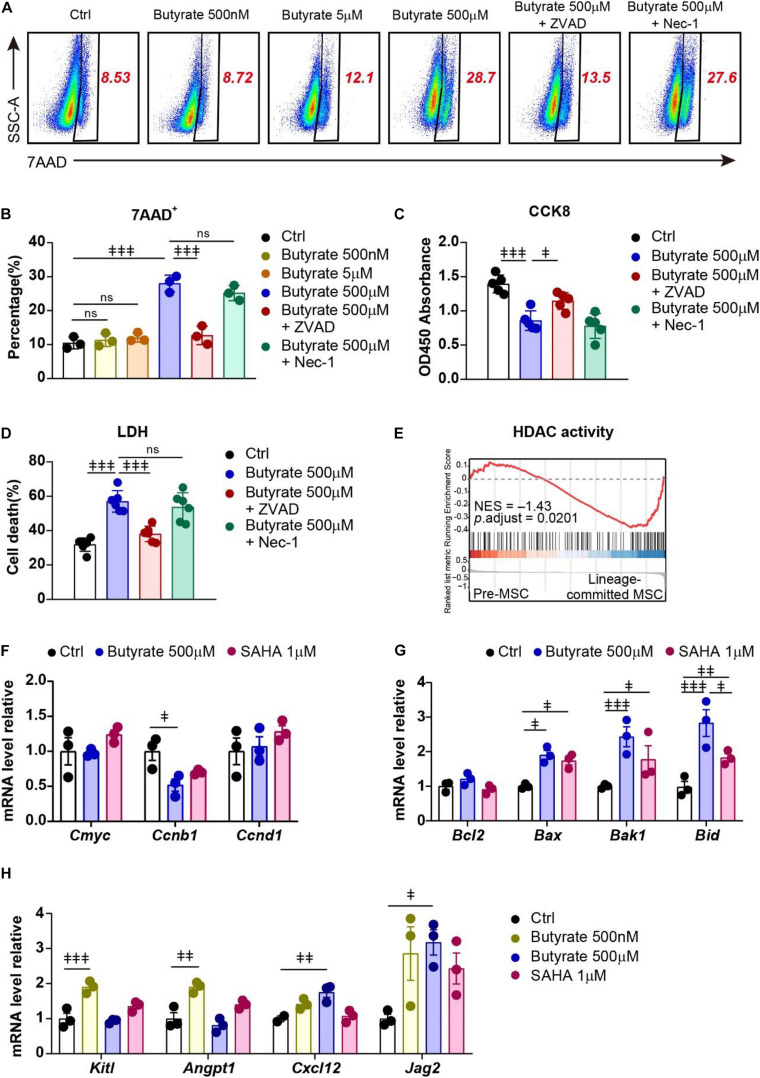
Supplement of butyrate triggers apoptosis but promotes HSC niche factor expression in MSCs. **(A)** Representative flow cytometry plots of 7AAD staining of MSCs under different treatments as indicated. The numbers in the plots denote the percentage of gated cells. **(B)** Percentage of 7AAD^+^ MSCs under different treatments as indicated. **(C)** CCK-8 assay showing MSC viability under different treatments as indicated. **(D)** LDH activity showing MSC death frequency under different treatments as indicated. **(E)** GSEA evaluating enrichment of genes associated with HDAC activity in pre- and lineage-committed MSCs. Normalized enrichment score and adjusted *p* value were calculated by permutation tests. **(F)** Quantitative RT-PCR of transcript levels of proliferation gene in MSCs with indicated treatments (normalized to control group). **(G)** Quantitative RT-PCR of transcript levels of apoptosis genes in MSCs with indicated treatments (normalized to control group). **(H)** Quantitative RT-PCR of transcript levels of HSC niche factor genes in MSCs with indicated treatments (normalized to control group). Repeated-measures one-way ANOVA followed by Dunnett’s test for multiple comparisons in **(B–D,F–H)**. ns, non-significant; 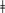
*p* < 0.05, 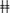
*p* < 0.01, 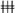
*p* < 0.001.

To investigate the potential mechanisms of butyrate treatment on MSCs, we performed 7AAD cell death staining to examine whether butyrate treatment influences their survival. We found that butyrate induced cell death in MSCs at a high concentration (500 μM, 2.67-fold increased) but not at low concentration (5 μM) or biological serum concentration (500 nM) (Figures 5A,B). We next asked whether butyrate induced MSC death through apoptosis or necroptosis and found that apoptosis inhibitor Z-VAD-FMK (ZVAD) but not necroptosis inhibitor Necrostatin-1 (Nec-1) completely blocked butyrate-induced cell death in MSCs (87.4%) (Figures 5A,B). Furthermore, we found that butyrate-induced cell growth inhibition (38.5% decreased) was also markedly rescued by ZVAD (53.6%) (Figure 5C). The consistent results were also observed in an LDH assay in which butyrate treatment increased the LDH activity (1.79-fold increased) and could be rescued by ZVAD (75.3%) (Figure 5D). These demonstrate that butyrate supplement attenuates the survival pathway and further triggers apoptosis in MSCs.

Butyrate is shown to regulate gene expression in blood cells through inhibition of HDAC (Song et al., 2011; Akimova et al., 2012; Arpaia et al., 2013). Interestingly, our scRNA-seq data revealed that lineage-committed MSCs had enriched HDAC activity (GO:0000118, GO:0004407, GO:0042826) compared to premature MSCs (NES = –1.43; Figure 5E). This indicates that butyrate might regulate MSC lineage commitment through influencing their gene expression network. Indeed, we found that butyrate treatment reduced *Ccnb1* expression in MSCs (48.1% decreased; Figure 5F), which might explain the butyrate-induced growth inhibition (Pines and Hunter, 1989). Butyrate treatment also induced upregulation of multiple apoptotic genes, including *Bax*, *Bak1*, and *Bid* (Youle and Strasser, 2008) (1.91-fold, 2.43-fold, and 2.83-fold increased, respectively; Figure 5G), which is consistent with increased apoptosis in butyrate-treated MSCs (Figures 5A,B). Furthermore, the downregulation of apoptosis genes upon butyrate treatment was in line with another HDAC inhibitor, suberoylanilide hydroxamic acid (SAHA) (Butler et al., 2002; Yin et al., 2018) (1.74-fold, 2.43-fold, and 1.83-fold increased, respectively; Figure 5G), suggesting that butyrate-induced MSC self-renewal potentially through HDAC inhibition.

As bone marrow MSCs play a pivotal role in maintaining HSCs through producing multiple growth factors (Crane et al., 2017; Pinho and Frenette, 2019), we next investigated how butyrate treatment influences MSCs in their HSC niche function. We found that butyrate significantly increased HSC niche factor expression, including *Kitl* and *Angpt1* at biological serum concentration (500 nM) (1.90-fold and 1.90-fold, respectively) and *Cxcl12*, *Jag2* at a higher concentration (500 μM) (1.75-fold and 3.17-fold, respectively; Figure 5H) (Greenbaum et al., 2013; Guo et al., 2017; Aprile et al., 2020). Interestingly, the HSC niche-inducing effect of butyrate was more robust compared to SAHA (*Kitl* 1.35-fold, *Angpt1* 1.40-fold, *Cxcl12* 1.07-fold, and *Jag2* 2.43-fold increased, respectively; Figure 5H). This indicates that metabolite butyrate is a robust HSC niche factor expression booster for MSCs during *in vitro* culture.

Overall, our data show that butyrate supplement dichotomously regulates MSCs in their self-renewal and HSC niche function potentially through altering the MSC metabolic status and inhibiting HDAC activity.

## Discussion

MSCs are present in all organs and tissues, and MSCs are a highly heterogenous subset (Uccelli et al., 2008). The heterogeneity of MSCs is closely related to their clinical utilities and also determines the barriers in transferring MSC capacities into the clinic (Costa et al., 2020). The functional heterogeneity of MSCs has been indicated in previous studies. For example, IL-17^+^ MSCs have enhanced antibacterial effects but with reduced immunosuppressive function compared with bulk MSCs due to altered NFκB-TGF-β signaling (Yang et al., 2013). Studies using RNA fluorescence *in situ* hybridization (FISH) (Cote et al., 2016) and fluorescent probes (Li et al., 2016) indicate that canonical markers are tenuously linked to the differentiated phenotypes, and it is difficult to use single markers to predict functional potential. Recent advanced single-cell studies have further explored the heterogeneity of bone marrow MSCs with distinct differentiation potential (Baryawno et al., 2019; Leimkuhler et al., 2020). Furthermore, researchers also identified an IL-10 regulated metabolically active mature adipocyte subtype from subcutaneous adipose tissue (Rajbhandari et al., 2019) and Runx2^+^/Gli1^+^ cells in the adult mouse incisor, which maintains Gli1^+^ MSCs (Chen et al., 2020).

In our work, we performed 10x scRNA-seq on bone marrow non-hematopoietic (CD45^–^, Ter-119^–^), non-endothelial (CD31^–^), and PDGFRα^+^ CD51^+^ MSCs. In line with previous studies (Pacini and Petrini, 2014; Baccin et al., 2020; Leimkuhler et al., 2020), we identified lineage-committed MSCs, including adipogenic, osteogenic, chondrogenic, angiogenic, and immunomodulating MSCs. Furthermore, we identified a pre-MSC population, which enriched prelineage commitment genes involved in protein transport, nuclear transport, and ribosome biogenesis pathways, such as *Rps24*, *Rpl35a*, and *Ndufb3* (Choesmel et al., 2008; Narla et al., 2011; Alston et al., 2016) and clustering at the root of the unsupervised pseudotime trajectory.

The metabolic profile determines the functional heterogeneity of MSCs (Costa et al., 2020). Genetic inhibition of mitochondrial complex III in human MSCs and murine adipocyte precursor cells impacts adipocyte differentiation (Tormos et al., 2011; Joffin et al., 2021). Glutamine metabolism regulates proliferation and osteoblast–adipocyte lineage determination (Yu et al., 2019). Among the diverse tissues from which MSCs could be isolated, the most common source tissue is the bone marrow (Yin et al., 2019). To meet the clinical requirement for MSCs in cell therapy, MSCs have to undergo a rapid cultural expansion and long-term cryopreservation, which are largely different compared with their biological microenvironment (Yuan et al., 2019). The metabolic profile determines MSC cell fate and heterogeneity (Pattappa et al., 2013; Moll et al., 2014; Costa et al., 2020). Interestingly, we discovered that pre-MSCs, despite the reduced expression of proliferation and differentiation genes, enriched genes in FA metabolic process compared with lineage-committed MSCs. This finding indicates that supplement of metabolites, such as FA in MSC during *in vitro* culture, may impact MSC functional heterogeneity.

Recent studies indicated that butyrate, one of the metabolites produced in the healthy intestinal lumen (Jacobi and Odle, 2012), enhances the effect of parathyroid hormone (PTH) to support bone formation (Fan et al., 2017; Li et al., 2020; Pacifici, 2020). Furthermore, butyrate promotes Treg cell regeneration and differentiation, which stimulates bone formation by activating Wnt signaling in osteoblasts (Arpaia et al., 2013; Furusawa et al., 2013; Tyagi et al., 2018). In our study, we discovered that supplement of butyrate suppressed the self-renewal capacity and differentiation potential toward osteoblasts and adipocytes in MSCs.

HDACs, a series of critical transcriptional cofactors modulating gene expression by deacetylating histones and transcription factors, participate in stemness maintenance, lineage commitment determination, cell differentiation, and proliferation as well as other activities in normal hematopoiesis (Akimova et al., 2012; Cortiguera et al., 2019). Homozygous deletion of HDAC3 in Prrx1-expressing cells reduced chondrocyte and osteoblast differentiation *in vitro* (Feigenson et al., 2017). HDAC inhibitors exhibit antitumor activity for multiple myeloma (Song et al., 2011; Santo et al., 2012) and B cell lymphoma (Cortiguera et al., 2019) by suppressing cells survival and differentiation and inducing apoptosis. Butyrate is one of the extensively studied HDAC inhibitors (Steliou et al., 2012). Butyrate blocks the activity of class I and II HDACs and increases histone acetylation globally in multiple types of cells, including CD8^+^ T cells, B cells, hepatocytes, and some tumor cell lines, such as MCF-7 (human breast cancer cells) and HCT116 (human colon carcinoma cells) (Candido et al., 1978; Davie, 2003; Fellows et al., 2018; Luu et al., 2018; Ji et al., 2019; Sanchez et al., 2020). In our work, we discovered that butyrate supplement reduced MSC proliferation and differentiation abilities at biological serum butyrate concentration without cell death. Butyrate treatment reduced the expression of proliferation gene *Ccnb1* but upregulated apoptotic genes, including *Bax*, *Bak1*, and *Bid*, which might explain the butyrate-induced growth inhibition. Furthermore, we found that lineage-committed MSCs enriched more genes associated with HDAC activity than pre-MSCs did. HDACs support cell growth for multiple tumor cells, including diffuse large B cell lymphoma, lung adenocarcinoma, and breast cancer (Gupta et al., 2012; Lapierre et al., 2016; Wang et al., 2016); therefore, HDAC inhibitors are clinically used for cancer treatment (Khan and La Thangue, 2012; Li and Seto, 2016). Our findings show that, unlike quiescent pre-MSCs, lineage-committed MSCs are more sensitive to HDAC inhibition, potentially due to their high proliferation potential.

Our data show that a high dose (500 μM) of butyrate treatment increased apoptosis in MSCs and also increased their HSC niche function. MSCs engulfed apoptotic bodies to enhance their differentiation ability and ameliorate the ovariectomy-induced osteopenia through activation of the Wnt/β-catenin pathway (Liu D. et al., 2018). Moreover, MSC apoptosis is related to enhanced osteoblast differentiation (Schaub et al., 2019), and apoptotic MSCs have enhanced immunosuppression activity when infused in patients with GvHD (Galleu et al., 2017; Burnham et al., 2020). These findings inspired us that the apoptosis of MSC might benefit their tissue regeneration or immunomodulation functions, which is consistent with our observation that a high dose of butyrate supplement increased apoptosis and enhanced their HSC niche function. Inhibition of HDAC2 and 3 promotes the proliferation of hematopoietic stem and progenitor cells (HSPCs) (Dhoke et al., 2016; Wang et al., 2020), but whether HDAC inhibition alters the HSC niche remains unclear. In our work, we demonstrate that *in vitro* supplement of butyrate-promotes HSC niche factor expression in MSCs, including *Kitl*, *Angpt1*, *Cxcl12*, and *Jag2*. As bone marrow MSCs produce SCF (*Kitl*), CXCL12 (*Cxcl12*) or Angiopoietin-1 (*Angpt1*) and endothelial cells secrete Jagged-2 (*Jag2*), which are critical for HSC maintenance (Greenbaum et al., 2013; Zhou et al., 2015; Guo et al., 2017; Comazzetto et al., 2019), our observation indicates that butyrate supplement may enhance HSC niche function potentially through inhibiting HDAC in MSCs. Overall, our findings indicate the possibility that the application of butyrate in MSC culture can amplify their HSC niche function and shed light on MSC treatments for patients with ineffective hematopoiesis and patients who underwent HSC transplantation.

## Data Availability Statement

The datasets presented in this study can be found in online repositories. The names of the repository/repositories and accession number(s) can be found below: GEO (GSE167035, https://www.ncbi.nlm.nih.gov/geo/query/acc.cgi?acc=GSE167035).

## Ethics Statement

The animal study was reviewed and approved by the Sun Yat-sen University.

## Author Contributions

JX performed the scRNA-seq data analysis and the functional assays, generated figures, and wrote the manuscript. QL, SX, and QX contributed to the scRNA-seq library construction and functional assays. YZ, YL, and LY contributed to the functional data analysis. JW performed and analyzed the scRNA-seq. LJ, LM, DL, and MZ supervised the project. All authors contributed to the article and approved the submitted version.

## Conflict of Interest

The authors declare that the research was conducted in the absence of any commercial or financial relationships that could be construed as a potential conflict of interest.

## Footnotes

1 https://www.ncbi.nlm.nih.gov/geo/query/acc.cgi?acc=GSE167035
