# Supplementary material for: Single-Cell Atlas Reveals Fatty Acid Metabolites Regulate the Functional Heterogeneity of Mesenchymal Stem Cells
Source: Front Cell Dev Biol. 2021 Apr 12;9:653308. doi: 10.3389/fcell.2021.653308 (PMC8075002; doi:10.3389/fcell.2021.653308)
Supplement: Supplementary file 1 [file Data_Sheet_1.docx]

**Supplemental Material for**

**Single-cell Atlas Reveals Fatty Acid Metabolites Regulate the Functional Heterogeneity of Mesenchymal Stem Cells**

Jiayi Xie^1,#^, Qi Lou^2,3,#^, YunxinZeng^1^^,#^, Yingying Liang^2,3^, Siyu Xie^4^, Quanhui Xu^5^, Lisha Yuan^5^, Jin Wang^5^, Linjia Jiang^4^, Lisha Mou^2^*, Dongjun Lin^1^*, Meng Zhao^1,2,5^*

Correspondence to:[zhaom38@mail.sysu.edu.cn](mailto:zhaom38@mail.sysu.edu.cn) (M.Z.), [lindj@mail.sysu.edu.cn](mailto:lindj@mail.sysu.edu.cn) (DJ.L.), [lishamou@gmail.com](mailto:lishamou@gmail.com) (LS. M.)

**This PDF file includes:**

Supplementary Figure 1


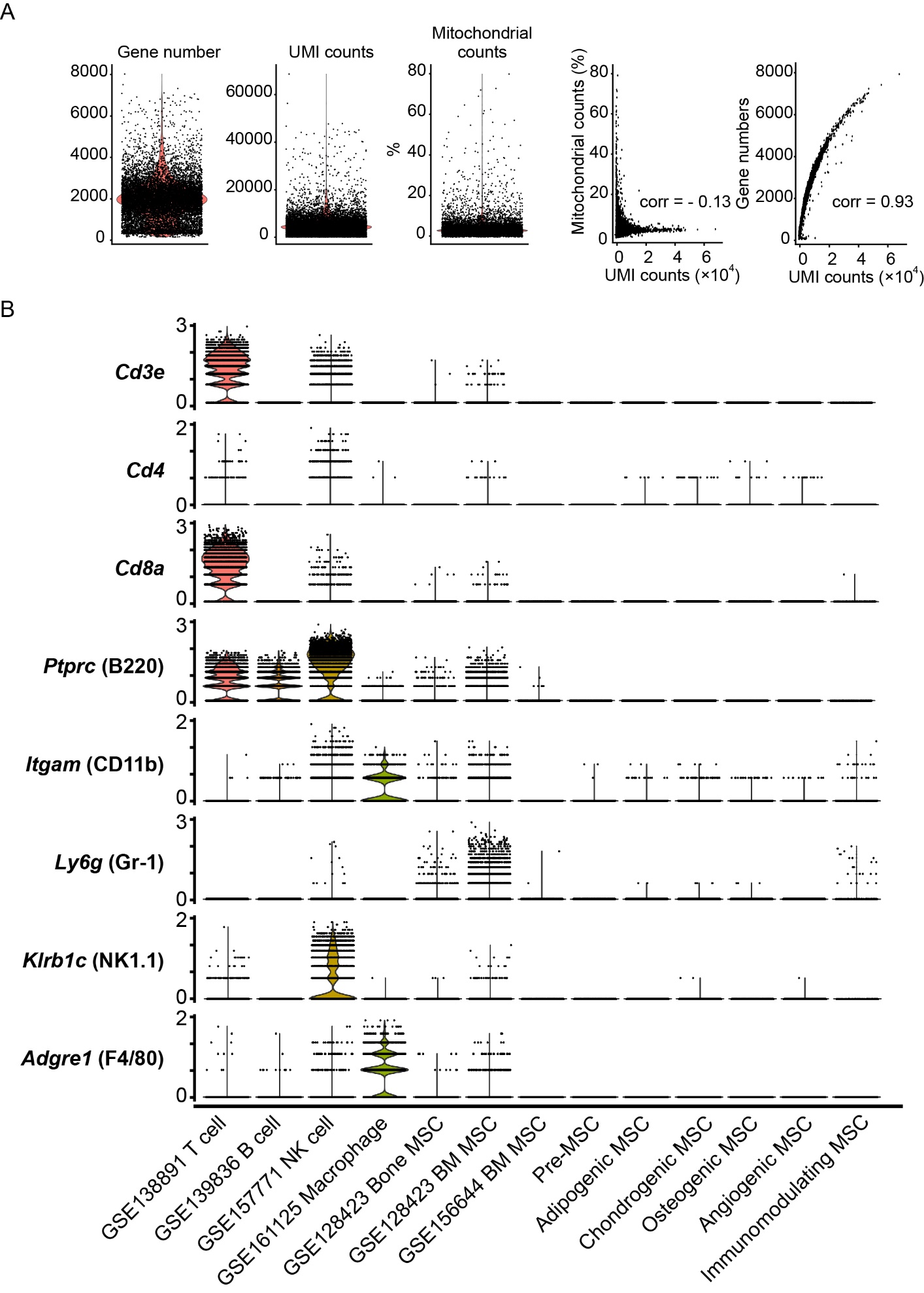


**Supplementary Figure 1. Quality control of single cell RNA sequencing data.**

(A) Violin plots showing the number of unique genes (Gene number), number of total unique molecular identifiers (UMI counts) and mitochondrial count fraction expressed in single MSCs after removed the cells which expressed *Ptprc*(CD45). Scatter plots showing the correlation between UMI counts and mitochondrial counts, gene numbers. corrs indicate Pearson correlation coefficients.

(B) Violin plots showing the indicated immune genes expression in published scRNA-seq on T cells (GSE138891), B cells (GSE139836), NK cells (GSE157771), Macrophages (GSE161125), MSCs (GSE128423 and GSE156644) and our MSC scRNA-seq data.
